# Patient experiences switching from in‐clinic to self‐administration of injectable contraception in two Western US states

**DOI:** 10.1111/psrh.12278

**Published:** 2024-07-04

**Authors:** Rebecca L. Newmark, Caroline C. Hodge, Grace Shih, Jennifer Karlin

**Affiliations:** ^1^ Department of Humanities and Social Sciences University of California San Francisco California USA; ^2^ School of Medicine University of California San Francisco California USA; ^3^ Department of Anthropology University of Pennsylvania Philadephia Pennsylvania USA; ^4^ Department of Family Medicine University of Washington Seattle Washington USA; ^5^ Department of Family and Community Medicine University of California San Francisco California USA

**Keywords:** contraception, hormonal implants/injectables, qualitative research methods, quality of care, united states

## Abstract

**Objective:**

We describe the experiences and preferences of women who switched from clinic‐administered intramuscular depot medroxyprogesterone acetate (DMPA‐IM) to self‐administered subcutaneous DMPA (DMPA‐SC) in the context of the COVID‐19 pandemic.

**Methods:**

We conducted interviews with women in California and Washington about their experiences with self‐administered DMPA‐SC. We interviewed women after their first or second self‐administered DMPA‐SC injection and conducted follow‐up interviews after their third or fourth injection. We performed both thematic and descriptive content analyses.

**Results:**

We completed 29 interviews with 15 women. Most participants (*n* = 10) were between the ages of 20 and 39 and the majority (*n* = 12) used DMPA primarily for contraception. Most (*n* = 13) described self‐administered DMPA‐SC as “very easy” or “somewhat easy” to use and reported greater convenience, decreased pain, fewer logistical and financial challenges, increased privacy, and improved comfort with injection compared to DMPA‐IM. Participants identified difficulties obtaining DMPA‐SC from pharmacies and safe needle disposal as barriers. Most (*n* = 13) would recommend DMPA‐SC to a friend and desired to continue self‐administration beyond the COVID‐19 pandemic. Participants recommended counseling all patients about this option alongside other contraceptive methods, and offering clinician supervision, if desired.

**Conclusion:**

Women who switched from in‐clinic DMPA‐IM to self‐administered DMPA‐SC during the COVID‐19 pandemic preferred the latter and intended to continue self‐administration. Self‐administration of DMPA‐SC should be routinely offered and easily accessible to patients.

## INTRODUCTION

Depot medroxyprogesterone acetate (DMPA) is a highly effective, reversible, progestin‐only injectable contraceptive that is also used to manage endometriosis, dysmenorrhea, and menorrhagia. Although initially formulated as an intramuscular (IM) injection, the United States (US) Food and Drug Administration (FDA) approved a subcutaneous (SC) version in 2004 in the form of a self‐contained syringe to be administered by a health care professional. Studies outside of the US suggest that patients who use DMPA‐SC find it less painful and report fewer side effects than those who use DMPA‐IM^1^, ^2^ and US studies have demonstrated the safety, feasibility, and acceptability of self‐administered DMPA‐SC,^3^, ^4^, ^5^, ^6^ including one that showed increased continuation of DMPA among those randomized to self‐administration (69%) compared to those randomized to clinic administration (54%).^7^ However, DMPA‐SC remains more expensive than DMPA‐IM and has not consistently been added to US formularies and clinicians across the US infrequently counsel patients about self‐administered DMPA‐SC.^5^

During the COVID‐19 pandemic, many clinics expanded access to self‐administered DMPA‐SC to meet public health aims to decrease in‐person visits. In April 2021, the Centers for Disease Control and Prevention (CDC) updated recommendations to include self‐administered DMPA‐SC as an additional method of contraception,^8^ echoing those of the World Health Organization (WHO),^9^ two systematic reviews,^4^, ^10^ and a 2021 expert panel.^8^ However, self‐administration of DMPA (IM or SC) remains off‐label in the US.^11^, ^12^

While our previous work confirmed a robust interest in DMPA‐SC self‐administration during the the COVID‐19 pandemic,^13^, ^14^ research on US patients' experiences of self‐administered DMPA‐SC and perceived benefits and challenges to this mode of delivery is limited.^15^ As such, we leveraged our prior implementation projects^13^, ^14^ to conduct longitudinal, in‐depth, qualitative interviews with patients to learn about their reasons for switching from DMPA‐IM to self‐administered DMPA‐SC, explore their experiences with counseling and use over time, and document their recommendations for counseling about this option. We aimed to understand patients' decision‐making to better support their sexual and reproductive health needs in the service of reproductive justice and equity.

## METHODS

The University of California, San Francisco (UCSF) Institutional Review Board approved all procedures prior to study initiation. At the start of the COVID‐19 pandemic, clinics affiliated with UCSF and University of Washington (UW) offered DMPA‐SC for self‐administration at home to patients already receiving DMPA‐IM in‐clinic. We recruited participants from four clinics across both institutions who had previously articulated an interest and intent to switch from the IM to the SC formulation of DMPA during the COVID‐19 pandemic.^13^, ^14^ None of these clinics closed during the COVID‐19 pandemic and all continued to offer DMPA‐IM in clinic.

**FIGURE 1:**
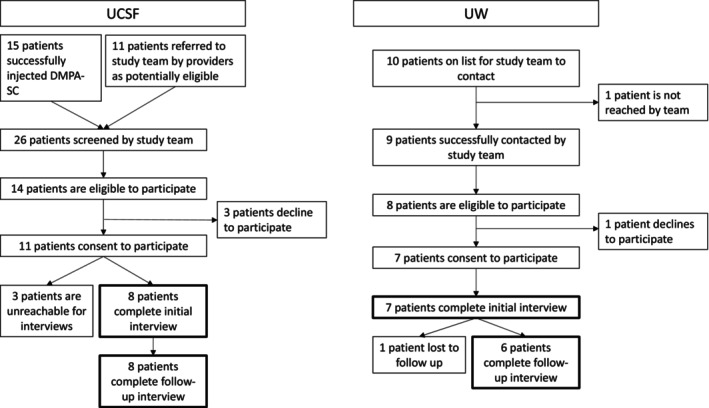
Recruitment of participants for interviews from two implementation studies at UCSF and UW of patients who switched from provider‐administered DMPA‐IM to self‐administered DMPA‐SC. DMPA: depot medroxyprogesterone Acetate; IM: intramuscular; UCSF: University of California, San Francisco; UW: University of Washington; SC: Subcutaneous.

Patients were eligible to participate if they were 18 years of age or older, spoke English or Spanish, and had successfully switched from DMPA‐IM to DMPA‐SC, as evidenced by at least one self‐administered dose of DMPA‐SC. We contacted potentially eligible patients by phone in June and July 2020 and asked whether they were interested in participating in two telephone interviews spaced 6 months apart about their experiences switching from in‐clinic DMPA‐IM to self‐administered DMPA‐SC. We attempted to contact potentially eligible patients a maximum of three times before leaving a general voice message with the contact information of the study team. During this initial outreach call, we took potential participants through an oral consent procedure and scheduled an initial interview at a time of their choosing. In all, we screened 36 patients and found 22 eligible to participate. Of these, 18 consented and we conducted initial interviews with 15 patients,14 of whom completed a follow‐up interview (see Figure 1).

We developed an interview guide of open‐ended questions about participants' previous experiences with contraceptives, initial interest in self‐administration of DMPA‐SC, experiences with counseling and injection, how experiences of self‐administration changed over time, and a priori themes around self‐efficacy, sense of control, and empowerment. The guide also included Likert scale questions of key outcomes from previous literature, including around ease of injection, pain during and after injection, intention to continue with DMPA‐SC, self‐efficacy, and likelihood that a participant would recommend DMPA‐SC to a friend (see Table 1).^1^, ^2^, ^3^, ^4^, ^5^, ^6^, ^7^, ^10^, ^15^ We followed the Patient Centered Outcomes Research Institute definition of “patient‐centered outcomes” as outcomes “meaningful and important to patients and caregivers.”^16^ We iteratively developed the follow‐up interview guide to include questions about discontinuation, if applicable, emerging themes from initial interviews, and changes in attitudes about and experiences with self‐administration since the initial interview. UCSF's Person‐Centered Reproductive Health Program's standing Patient Advisory Committee provided feedback on the interview guide, which we also translated into Spanish. At the end of each interview, we administered the new general self‐efficacy scale, a validated 8‐item scale that has demonstrated high reliability and predicts specific self‐efficacy for a variety of taskes in various contexts in multiple populations, to assess each participant's sense of general self‐efficacy.^17^ Each item on the scale is scored from one to five for a score range of 5 (low) to 40 (high).

**TABLE 1:** Demographics of patients at initial interview who switched from provider administered DMPA‐IM to self‐administered DMPA‐SC (*N* = 15).

Participant Characteristics		*n* (%)
Age (years)	Median (range)	35 (25–55)
	20–29	5 (33)
	30–39	5 (33)
	40–49	3 (20)
	50–59	2 (13)
Gender	Woman	15 (100)
Race/Ethnicity*	Black/African American	2 (13)
	White/Caucasian	6 (40)
	“Mixed race”	1 (7)
	Indigenous	2 (13)
	Latina hispanic	4 (27)
Education	Less than high school	4 (27)
	High school/GED	3 (20)
	Some college	5 (33)
	Vocational degree	1 (7)
	Bachelor's or higher	2 (13)
Using DMPA primarily for contraception	Yes	12 (80)
	No	3 (20)
Has child (ren)	Yes	10 (67)
	No	5 (33)
Previous experience with needles	Yes	9 (60)
	No	6 (40)
Approximate duration of DMPA‐IM (months)	Mean (range)	59.9 (10–180)
Number of DMPA‐SC injections before initial interview	1	7 (47)
	2	8 (53)

Abbreviations: DMPA: depot medroxy‐progesterone acetate; GED: graduate equivalency degree; IM: intramuscular; SC: subcutaneous.

* Patient‐reported.

We interviewed 15 participants at UCSF (*n* = 8) and UW (*n* = 7) at two time points, the first as close to the first injection as possible and the second after the third or fourth injection, with a minimum of 6 months between interviews. RN and CP conducted initial interviews with English‐ and Spanish‐speaking participants, respectively, between August and December 2020 and follow‐up interviews over the phone between February and May 2021. JK provided guidance throughout the interview process. Participants received a USD50 gift card after completing each interview, up to USD100 total. We audio‐recorded all interviews and hired a professional service to transcribe all interviews and translate the Spanish language interviews into English.

Data analysis started concurrently with data collection. We used a deductive‐inductive directed content analysis approach described by Hsieh and Shannon to analyze our transcripts, coded for a priori and emergent themes,^18^ and managed our data with ATLAS.ti software. All members of the research team iteratively developed a codebook beginning with an initial review of transcripts. Subsequently, RN and CH independently coded the transcripts using the final codebook, which the team assessed for an inter‐rater reliability of 90% halfway through data analysis. We report our results from the descriptive content analysis by domains of inquiry, following the Standards for Reporting Qualitative Research, as described by O'Brien and colleagues.^19^

## RESULTS

### Participant characteristics

As presented on Table 1, participants ranged in age from 25 to 55 with a median age of 35 and all identified as cisgender women. Six (40%) identified as white, two (13%) as Black/African American, and seven (47%) as “mixed,” Central American Indigenous, or Latina/Hispanic. Three participants (20%) completed a vocational or bachelor's degree and most participants (*n* = 10) had at least one child at interview. Nine participants (60%) had previous experience with self‐injection or assisting others with injections. Four participants (26%) discontinued DMPA‐SC between the initial and follow‐up interviews, one because she was no longer sexually active, one because she preferred clinic administration, and two because they had difficulty obtaining DMPA‐SC prescriptions at their pharmacy. Clinic records indicate that the participant who did not complete a follow‐up interview continued with self‐administration of DMPA‐SC. No participants reported becoming pregnant between interviews.

### Interest in self‐administered DMPA‐SC

Most participants indicated that they initially switched from DMPA‐IM to DMPA‐SC because of a desire to social distance during the COVID‐19 pandemic and all but one (*n* = 14) continued to use DMPA‐SC because they preferred it to DMPA‐IM. These participants felt strongly that DMPA‐SC self‐administration should continue to be an option beyond the pandemic. When asked who should use DMPA‐SC, many participants talked about “busy” people—busy moms, busy working professionals, busy college students. Star, a mixed race 29‐year‐old woman with some college experience from Washington state remarked in her first interview:

I just feel like it should be an option permanently just because there is a lot of moms and just women who have really busy lifestyles, and it is really hard to make an appointment sometimes, especially when you are on a certain schedule. You have to get in when you have to or otherwise you miss, and you have got to start all over. It just makes it a lot easier. I feel like a lot of women would really appreciate doing it themselves.

In her second interview, Star elaborated when we asked her about if she would recommend this to a friend:

I think of us‐especially my friends, we are all busy. We have busy jobs, busy lives. Trying to fit in an appointment to remember to go get your shot is just one more thing that you have got to try and fit in. If you can get it at home, do it at home. There is been a couple of my friends that I have referred to my doctor. They now do it at home too, and they love it. For some of us, for me to go to my doctor's office, it is a half‐an‐hour drive each way plus whatever time there. I drive 20 miles out of my way to go and see my doctor. It is easier to drive 5 min away from my house, pick it up at my pharmacy, and come home and do it.

Others noted that they would recommend it to “anyone” and almost everyone (*n* = 13, 93%) said they would recommend it to their friends when asked at follow‐up.

Those who had previously self‐administered injections of other drugs mentioned that their experience was helpful in feeling comfortable switching from provider‐administered to self‐administered DMPA. Jude, a Black 53‐year‐old woman from San Francisco with some high school education said in her first interview, “I inject myself every tuesday with Ozempic. It is to lower your blood sugar and everything. So, I have to inject myself every Tuesday with that anyway. It is a little bitty needle that I have to use. So that kind of prepared me for the Depo [DMPA].” Lynn, a white 38‐year‐old woman with a Master's degree from Washington state noted that, while she did not have firsthand experience with needles, seeing others inject insulin gave her confidence, “I went to an office lunch about a year ago and one of my coworkers just popped out a needle and stuck herself with it at the table like it was no big thing. So, I was like ‘well if she can do that, it cannot be that bad.’”

While participants acknowledged that people with previous experience administering injections, like those with chronic health conditions, might be especially good candidates for self‐administration of DMPA‐SC, they emphasized that everyone deserved the option to do it. As Steph, a white, 42‐year‐old woman from Washington state with some college experience said in her second interview:

I think it is just I have never been one that is apprehensive about needles, but there are people that are very apprehensive. I think if they were given the chance, then they will realize, hey, this is not so bad… It is easy, and I think more people need to have access to things like that where they do not have to go to the doctor to have things done.

Participants further suggested that caregivers or other people in the community could administer the injections if they did not want to do it themselves.

### Counseling and awareness about self‐administration of DMPA‐SC

Most participants suggested that self‐administered DMPA‐SC should be part of routine contraceptive counseling and encouraged clinicians to highlight the convenience, privacy, and ease of use. In her second interview, Lynn asserted, “I think this would be a really good option if you were somebody who was either very private or, for some reason, you wanted to take a form of [contraception] that, say, people in your household did not need to know about in any way, shape, or form. This would be very easy.” Participants felt strongly that everyone should have the option to self‐administer, as Jude vehemently expressed in her second interview, “Give them a choice. Explain the difference between the two, and then give you a chance to weigh out the pros and cons, you know?” While some were unsure how to advertise the option of self‐administered DMPA‐SC, multiple participants suggested social media. Participants also recommended in‐clinic pamphlets to support clinician counseling.

Few participants received in‐person counseling about DMPA‐SC during its implementation in the COVID‐19 pandemic. Star positively described her experience of in person counseling during the first interview, “My doctor was really into showing me everything. She made sure I knew how big the needle was, how it was going to hurt, and basically how to be clean with it. I was pretty comfortable with trying it myself even if I did not have experience, and it was really easy.” While many expressed a desire for in‐person training, most of our participants received counseling by phone and supplemental materials (e.g., pamphlets/reading materials and/or links to how‐to videos on self‐injection) by email. Several participants suggested that clinicians offer a non‐mandatory, supervised first injection to allow patients to ask questions during the process.

When asked how to inform patients about DMPA‐SC without being coercive, participants consistently responded that it should be presented alongside all other contraceptive options, without targeting certain populations. For example, Lynn reflected in her second interview,

To me, it is more of just making information available to a wide variety of folks and in the way that they need it. So, if I need something in a language other than English, making sure that I get all my questions answered and all the information I need in order to make that decision in the language that I speak and understand. Or, if I'm somebody who is, say, of a community—like if I'm non‐binary or transgender or something like that—in a way that makes sense for where I'm coming from so that I can make those informed decisions for myself based on where I'm at.

None of our participants felt personally pressured to use DMPA‐SC, and most were unaware that marginalized populations have at times been forced, encouraged, or dissuaded by clinicians to use particular contraceptives.^19^, ^20^ Luz, a 25‐year‐old Latina patient from San Francisco with some college remarked in their first interview, “I did not feel, like, forced because they told me, you know, like, okay, if at any given moment you feel like you do not want to do it, just call us back. We will schedule an appointment. You do not have to do it if you do not want to or if you do not feel safe.” Participants highlighted the importance of non‐coercive contraceptive counseling practices in both sets of interviews.

### Obtaining and disposing of DMPA‐SC

Some participants reported difficulties obtaining DMPA‐SC at the pharmacy due to lack of insurance coverage and high co‐pays, pharmacist’ reticence to dispense the drug due to its FDA label specifying clinician‐only administration, or suspicions that the injection had not been ordered correctly. Luz described availability as a barrier, leading her to plan ahead with the pharmacy to avoid missing a dose, “I would say the only hard part of [doing the shot] was working along with the pharmacy, because at times they were out of stock. So, I just had to make sure that I called a week in advance before my shot was due so I did not run into a problem of them telling me, ‘Oh, we do not have it. You have to wait another week to get it,’ or, you know, even just a couple of days.” Participants suggested that clinicians order multiple refills of DMPA‐SC, and that users be reminded to pick up the medication a week or two before the next dose needs to be administered.

Two of the four participants who discontinued DMPA‐SC after the initial interview did so because of administrative hurdles. Even though she planned to continue at home injections, Lynn explained during her second interview that she was unable to pick up the prescription from her pharmacy. After returning to the pharmacy twice to pick up the prescription only to find that it was not in stock, Lynn gave up and scheduled a clinic appointment. She explained, “I much prefer doing it at home, it is very simple. The first time, I had to wait a few days for when they put in the order from the doctor's office to the pharmacy for them to get it in stock.” Several other participants echoed this experience, noting that they needed to call ahead to the pharmacy to ensure their medication was available.

Participants found needle disposal a challenge. While most received counseling about how to dispose of their needles, and most shared that they would have liked to have received a sharps container, many did not receive one when they picked up their DMPA‐SC. One participant independently purchased a sharps container to keep at home, while others took them back to the pharmacy for disposal. Some participants reported throwing used needles in their garbage, or placing needles back in the medication box before throwing them away like Jude explained during her first interview, “All my medications come in Walgreen's [a pharmacy chain retailer] bags. So, I put it back in the container that I took it out of, I put the top back on the needle part, the cover back on the needle that it came with, I put it back in the container, I put it in a Walgreen's bag, and I rolled it up, and I threw it in my kitchen garbage.” Others tried more creative solutions, as Star described, “I put it in a little Tupperware container. The needle comes with a cover, so when you are done, you just put that cover over it, and no one can poke themselves. I use that, but I also put it in a little Tupperware container because you are not supposed to throw your needles in your trash can.”

### Self‐administration of DMPA‐SC

In the initial interview, most participants described DMPA‐SC self‐administration as “very easy” or “somewhat easy” to use (see Table 2), which many found surprising. Several participants experienced confusion over where to inject DMPA‐SC because DMPA‐IM is injected in the arm, and DMPA‐SC is often injected in the abdomen or the thigh. Indeed, two participants shared that they initially injected DMPA‐SC in their arm. Both participants who described DMPA‐SC as “difficult” to use at the initial interview shared that it got easier over time, describing it as “very easy” at the follow‐up interview. Several participants shared that it was easier to do the shot each time and that it became “no big deal.”Indeed, participants repeatedly invoked ease of use as one of the benefits of DMPA‐SC at follow‐up. Brooke, a 35‐year‐old, white participant who had completed her general educational development exam (GED) said in their first interview, “It really was really easy, yeah. It was kind of weird that it was, like, so easy. Like because I do not know. It was kind of trippy. It kind of freaked me out a little bit, and I was, like, oh, this is weird. Like I get to give myself a shot? Weird. But, yeah, and then I just made the joke with my mom and I just did it.” This initial trepidation followed by increased comfort self‐administering injections was common among participants.

**TABLE 2:** Key outcomes about experiences of self‐administration of DMPA‐SC reported by initial (*N* = 15) and follow‐up interviewees (*N* = 14).

		Initial interview participants (*N* = 15)	Follow‐up interview participants (*N* = 14)
Outcome		*n* (%)	*n* (%)
Ease to inject	Very easy	8 (53)	10 (71)
	Somewhat easy	5 (33)	0 (0)
	Somewhat difficult	1 (7)	0 (0)
	Very difficult	1 (7)	0 (0)
	N/A (discontinued after initial interview)		4 (29)
Pain during shot	Not painful	6 (40)	8 (57)
	A little painful	9 (60)	2 (14)
	Very painful	0 (0)	0 (0)
	N/A (discontinued after initial interview)		4 (29)
Pain after shot	Not painful	12 (80)	9 (64)
	A little painful	3 (20)	1 (7)
	Very painful	0 (0)	0 (0)
	N/A (discontinued after initial interview)		4 (29)
Intend to continue with self‐administration	Yes	14 (93)	10 (71)
	No	1 (7)	0 (0)
	N/A (discontinued after initial interview)		4 (29)
Would recommend self‐administered DMPA‐SC to a friend*	Yes		13 (93)
	No		1 (7)
Self‐efficacy	Mean (Range)	34.1 (30–40)	33.6 (27–40)

Abbreviations: DMPA: depot medroxy‐progesterone acetate; SC: subcutaneous.

* Probed only during the follow‐up interview.

A few participants noted a fear of needles as an obstacle they had to overcome to use DMPA‐SC at home. Importantly, those who reported this fear either found workarounds to minimize their handling of the needle—such as recruiting a trusted friend or family member to help them with injection—or expressed a desire to work through their fear as a self‐improvement exercise, like Jude explained, “I was actually very proud of myself. I thought that I was not going to be able to do it. But the needle was only, like, a half an inch. It was not big at all. And when I did it, I felt accomplished actually. I actually did it myself. So it—it felt great.” Dev, a 28‐year old white participant from Washington similarly described in her first interview, “I think [I] was just like nervous about, like, poking myself. Like, you know, like getting like the courage to just, like, stab. But the needle was so tiny, so I do not know why I was thinking that. Because once I did it, it was fine.”

All participants rated DMPA‐SC as either “not painful” or “a little painful” to administer and many noted that it was less painful than the DMPA‐IM injections they received in clinic. Some participants attributed the decreased pain associated with DMPA‐SC to different injection sites (i.e., arm vs abdomen), while others, like Maria, a 42 year old San Franciscan participant with a 6th grade education, attributed decreased pain to self‐administration, “I'm going to say [that doing the injection at home was] less sore because I did it myself and I was more gentle to myself.” While many participants experienced soreness following DMPA‐IM injections in clinic, they did not experience it following DMPA‐SC injections at home, “Actually, it was more sore at the doctor's [office]. I feel like they gave us smaller needles for the at‐home injections than they did with the ones in the clinic. I'm not 100% sure, but I mean, I have gotten a Depo shot enough times in the clinic to know that that needle is not the same,” stated Jill, a 30 year old, white Washington participant with a high school education, during her first interview.

Participants reported increased ease of self‐injection over time, even those who initially described challenges in their first administration. As Dev said in her follow‐up interview, “I definitely got better. I learned how to position myself better, like, sit better, because I give it in my stomach. And I just learned exactly how I need to sit, and then I need to wait a second before I get up, or it squeezes back out. It is way easier now. I learned the pressure to put, like how much force I need and pace‐like, how quickly I should do it. I have it down pretty good now.”

Only one participant experienced side effects when she switched from DMPA‐IM to DMPA‐SC. She reported a headache after the first self‐injection of DMPA‐SC and noted that she had never experienced any headaches following DMPA‐IM injections in‐clinic. The remaining participants experienced no change in side effects between DMPA‐IM and DMPA‐SC. For those who were using DMPA to manage heavy menstrual bleeding rather than as a contraceptive (*n* = 3), none experienced a change in expected menstrual bleeding following the switch from DMPA‐IM to DMPA‐SC.

When asked if they encountered any surprises in the self‐injection process, several participants noted that they needed to push harder to inject the medication than they expected, and some were worried that they did not inject all the medication. Lynn explained in her first interview:

I had to press harder with a continual press than I thought I would. So, when I first did it, I kind of pressed down on the plunger thought it would just go in, and it would take like two seconds and be over. And I kind of stopped in the middle of it and had to sort of, okay, that did not all go in. And then restart it again. Like in my mind, I thought it was just like a quick like one, two, three, you are done. It is more of a, like a one, two, three, four, five, six, seven, eight, nine, ten kind of thing.

Similarly, Star said during the first interview, “The most difficult part was just the liquid. I was not really aware of how thick the liquid was. I thought it was just going to be a simple poke and easy, like, to push it in. But I had to really like push it in.” Remembering when to administer the injection was also challenging for some participants. Several noted using systems like phone app reminders or physical calendars to remember when to do the shot and others “just [tried] to remember.”

### Patient‐centered outcomes

At both initial and follow‐up interviews, most participants intended to continue using DMPA‐SC at home. Of the four who discontinued DMPA‐SC, one participant shared at the first interview that she would continue DMPA‐SC until she could resume getting injections in the clinic. She explained that receiving the injection in clinic was more convenient since she had to go there regularly for other health concerns. Only one participant did not intend to continue DMPA‐SC, or any other contraceptive method, after the initial interview due to a change in her relationship status and sexual activity.

#### Privacy

Privacy emerged in the initial interviews as a benefit of self‐administration, and was further highlighted in follow‐up interviews. Some participants explained that, while the clinic setting never bothered them, getting to administer the shot in the privacy of their own home was a more pleasant experience. Further, participants identified privacy as a possible “selling point” for DMPA‐SC when providers discussed contraceptive options with patients.

As opposed to conceptualizing privacy in the context of contraceptive counseling, participants primarily reported concerns about the over‐abundance of impertinent questions clinicians asked during contraceptive consultations. Multiple participants talked about their sexuality being “on display” or otherwise made public by getting DMPA‐IM injections in‐clinic. Several related experiences of having unsolicited and unwelcome comments made about their sexuality or sexual activity when getting the shot in‐clinic. Angel, a Latina 25 year old from San Francisco nursing student described experiences that felt intrusive and unnecessary in clinics:

I definitely like it better than going to the hospital, because usually at the hospital, they just ask you a bunch of questions. Like, some of them, I do not think are necessary questions. Like they will ask do I have a boyfriend? Do I have a husband? Do I have kids? And I'm like, technically, I'm assuming you could see that in my record if I had any kids or I was married or, for whatever reason, other information…like they are judging me because I am young or you are judging me because, you know, if you ask me, like, oh, are you married and I tell you no, like, I just feel like—just that itself, it is very different…So, I like that aspect of it. I do not have to give anybody any explanations like, “Oh. Why are you getting the shot?”

Steph stated in her second interview how doing the shot at home allowed her more privacy:

I do not have to go in and do the, “You need to bare your tush, and we are going to give you a shot here, and when is the last time you had sex,” or “Are you sure you are not pregnant?” I do not have to tell anybody that. I do not have to tell them. Hey, I'm 42, and I'm not having sex. I'm a happy, crazy, old cat lady. But, it is nicer to just get it. Do not even get questioned at the pharmacy. They fill it, and, “Hey, you are ready to go.” “Cool.”

Some participants also identified that the increased privacy associated with home administration could be a possible asset for individuals who need or prefer to keep their contraceptive use private or hidden from others, even if that was not something they preferred or needed themselves.

#### Convenience

All participants agreed that self‐administered DMPA‐SC is more convenient than visiting a clinic for DMPA‐IM and saves time spent scheduling an appointment, traveling to a clinic rather than a local pharmacy, and waiting to see a clinician. Several shared that arranging childcare for appointments is a logistical and financial challenge obviated through self‐administration. Finally, participants reported that self‐administration allows more flexibility in scheduling a time to inject, whether right before bed, on lunch breaks, or while working from home. Lynn remarked during the second interview:

It is just so much more convenient. At least at my doctor's office, you have to get to the clinic, check in with the front desk, wait in the waiting room, wait for whoever's giving you the shot. Sometimes you have to weigh in on the scale, get your blood pressure taken. So, it feels like going to the doctor every 3 months whereas when you have the at‐home prescription, literally the only thing I have to do besides giving myself the shot is to either go to the pharmacy and pick up my prescription or have somebody else pick it up for me.

#### Impact on self‐efficacy and confidence

We initially hypothesized that DMPA‐SC self‐administration would lead to an increase in self‐efficacy between the initial and follow‐up interviews. However, participants demonstrated little difference between self‐efficacy scores at initial interview and follow‐up interview (see Table 2). But, most participants agreed that they felt more capable, comfortable, and confident in administering the shots over time. One participant did not feel self‐injection became easier due to the long interval between shots and thought she would become more comfortable if the shots were more frequent (i.e., weekly); however, she was not deterred from continuing self‐injection of DMPA‐SC at home.

While we expected participants to discuss at‐home administration of DMPA‐SC as “empowering,” most describe feeling capable and proud. Indeed, only Jude specifically referred to her experience as such:

I'm in a wheelchair. I have serious severe back issues. I got a lot of issues—diabetes, high blood pressure. So, a lot of the times I feel defeated because of all of my health issues. Like I'm 53 years old. I cannot walk hardly. I can barely do much of anything myself. And, so, when I was able to do that shot I felt—when I was able to do it myself… it did make me feel great that I was actually able to do something without having assistance like I have to have 99% of the time, and that can be a sense of shame, you know, at times. So, it was very empowering. It makes me feel very much more independent and that “I'm actually doing something for myself, to help one of my health issues. So, I feel very, very confident, and very empowered.”

Multiple participants reported feeling confident because “my doctor is trusting me to do this for myself,” and creating opportunities to exercise their bodily autonomy. Steph remarked, “This is something that I would normally have to go in for, and now they are trusting me to do it myself. They have given me the tools to do it myself. I think it feels a little bit better knowing that I don't have to go to the doctor to get the shot, that they trust that I can do myself.”

While most participants agreed that this increased sense of capability did not directly connect to other areas of their lives, they acknowledged that the feeling in this part of their life was meaningful and significant to them as Dev said in her second interview:

Interviewer: okay. And has the experience of doing Depo at home made you feel more or less capable in any other areas of your life?

Respondent: I guess it could, because you just feel capable in general. Like, “Well, I can do this, so, like, I could probably,” you know, it just boosts your confidence all around. To, like, do whatever. It is like one more thing to add to the list of things that I can do by myself, I guess. Add it to the list.

Participants expressed this sense of capability in terms of feeling in control of their own health, as Steph explained:

Just knowing that I can handle it, and I can do it, and I do not have to go to the doctor to get it and that it is feasible now—more feasible now to take more control of your health outside of the doctor's office. You do not have to go to the doctor for this or have to go to the doctor for that. They are opening the channels of people being able to take a little bit more control of their health. It is like, okay, I got this. They trust me enough that I can do this. It feels good.

## DISCUSSION

This is the first longitudinal study that characterizes the experiences of US patients who switched from in‐clinic administration of DMPA‐IM to at‐home administration of DMPA‐SC. While our participants overwhelmingly articulated positive experiences with both formulations and methods of injection, virtually all participants expressed a preference for self‐administration of DMPA‐SC. These results echo one recent article which found that patients appreciated the option and found self‐administration empowering, despite initial hesitation and logistical challenges obtaining DMPA‐SC.^15^ Only four participants discontinued use of DMPA‐SC after the initial interview; all other participants continued DMPA‐SC and had plans to continue indefinitely. Most participants found DMPA‐SC easy to administer and less painful than the DMPA‐IM injections they previously received in‐clinic, and would recommend it to a friend. Participants identified high co‐pays and a lack of insurance coverage, syringe disposal, and a lack of availability of DMPA‐SC at pharmacies and/or a reluctance among pharmacists to dispense the drug as barriers.

Participants from our study made clear that all patients seeking contraceptive care should be counseled about this option and that there was not a certain stereotype of person that may be interested in or capable of self‐administering DMPA‐SC. Participants recommended the following to improve access to self‐administered DMPA‐SC: (1) routine inclusion of DMPA‐SC in all contraceptive counseling; (2) provision of a sharps containers when dispensing DMPA‐SC; (3) education tailored to patient preference and optional clinician observation of first self‐injection; (4) discussing patient‐centered benefits like privacy, improved ease of use over time, and convenience during options counseling; (5) discussing methods to remember to administer regular injections; and (6) streamlining pharmacy orders, insurance coverage, and reimbursement.

Participants also expressed a strong desire to be supported in their contraceptive decisions to allow them to care for themselves, their families, and their communities, a central tenet of the reproductive justice movement.^20^, ^21^, ^22^ Although their decisions about contraceptive choices were dynamic and changed given their relationships and their life circumstances, our participants described wanting to be able to choose the “easiest” or “most convenient” contraceptive methods to fit their lifestyles. This speaks to the importance of equity in contraceptive counseling because the majority of DMPA users in the US are Black and adolescents.^23^ It is a form of structural oppression that self‐administered DMPA‐SC is not routinely offered in contraception counseling, readily available at pharmacies, or covered by insurance. This point is echoed by Burlando and colleagues, who argue that making DMPA‐SC available for self‐administration is an equity issue.^24^ Thus, if health care providers do not offer it and payors do not make it affordable to all as standard practice, we are worsening inequities by withholding a convenient method of contraception from those who are most likely to use it.

Moreover, one of the central tenets of patient‐centered care is prioritizing individual patient's needs, desires, and goals.^25^ This framework has been operationalized into a reliable and valid performance measure of patients' experiences of patient‐centered contraceptive counseling that correlates with patient satisfaction called the Person Centered Contraceptive Counseling (PCCC) scale.^26^ The four questions included in this measure assess if the patient felt their provider respected them, let them say what mattered to them about their contraceptive method, took their preferences about contraception seriously, and gave them enough information to make the best decision about their contraceptive method. Our participants reiterated the importance of these four facets of counseling, suggesting that patient‐centered contraceptive counseling should be inherent to contraceptive care, including for self‐administered DMPA‐SC. That our participants felt more capable because their health care provider trusted them suggests that person‐centered contraceptive counseling may increase patient capability, autonomy, and satisfaction. By respecting and honoring patients' contraceptive choices, providers can improve patient outcomes, the patient‐provider relationship, and quality of care.^27^, ^28^, ^29^, ^30^, ^31^ Next steps for studying this relationship between offering self‐administration of DMPA‐SC could include measuring if the PCCC scale improves with different counseling techniques for offering and teaching about DMPA‐SC for self‐administration.

As DMPA‐SC becomes more widely available due to increased provider awareness and greater insurance coverage,^32^ it is important that clinicians providing contraceptive counseling are well‐informed about the medical evidence and patient perspectives to better counsel patients. Also, DMPA‐SC must be integrated into medical school curricula and continuing education and training to ensure that current and future providers are prepared to integrate this contraceptive option into their practice. Moreover, participants' experiences with pharmacists' confusion and hesitancy about dispensing the medication suggest designating self‐administrated DMPA‐SC as “off‐label” can create barriers to access. The FDA should consider revising the DMPA‐SC label to include self‐administration to alleviate the reticence among physicians, pharmacists, and other health care providers to counsel patients on self‐administration and thus decrease access barriers for patients.

Notably, most participants found self‐administered DMPA‐SC “very easy” or “somewhat easy” to use despite receiving no in‐person training prior to their initial shot. This differs considerably from standard practice in many low‐ and middle‐income countries, where individuals typically receive in‐person training before being “certified competent to self‐inject” and given medication to administer at home.^1^, ^2^, ^5^, ^9^ While our participants' ease despite receiving no training may be attributable, in part, to previous injection experience in our sample (60%), we believe it speaks to the fundamental ease of use of this method and suggests that we may be too prescriptive and paternalistic when recommending that individuals receive in‐person training and become “certified” in order to inject safely at home. Our participants' success self‐administering injections and their satisfaction with counseling without an in‐person visit suggests that a person‐centered contraceptive counseling framework, including optional in‐person training, could improve contraceptive access for individuals who do not live near a clinic or have other obstacles to attending such trainings.

### Limitations

The primary limitation of this study is its small sample size and that we recruited participants from four clinics in two Western states; thus, our sample does not mirror the diversity of demographics nor experiences throughout the US. Additionally, we only interviewed women who were previous DMPA‐IM users, so our results cannot be applied to populations that have not previously used DMPA. Also, our sample only includes patients who switched to self‐administered DMPA‐SC during the COVID‐19 pandemic when clinic resources were limited. While the pandemic generally altered contraceptive access across the US, the clinics from which we recruited remained open and had wide availability of contraceptive injections during this time. Further studies of all contraceptive users who switch to DMPA outside of a pandemic would add to our understanding of how DMPA‐SC fits into the panoply of contraceptive methods available.

All four of the researchers are cis‐gendered women in the medical profession (two doctors with specialty training in family planning and two medical students). Three of the four authors are trained anthropologists. Reflecting on our positionality, we recognize that all of us are supportive of care which offers the most possibilities for contraceptive autonomy and choice; this may skew the results to being more supportive of patients choices about their health care. While the open‐ended and inductive nature of ethnographic semi‐structured interviews facilitated, for instance, the nuanced senses and entangled meanings of empowerment, trust, capability, and ease that marked our participants' experiences of DMPA‐SC, the context‐dependence of ethnographic interviews can compound the difficulty in drawing general conclusions from an already small sample size. In all modalities of research, the preconceptions of the researchers can skew the results.

## CONCLUSION

We found that patients who switched from clinician‐administered DMPA‐IM to self‐administered DMPA‐SC were satisfied with this contraceptive method and our results suggest that it should be offered more broadly. DPMA‐SC is private, convenient, and easy‐to‐use and could play an important role in enhancing reproductive autonomy and equitable contraceptive care in the US. Patient‐centered contraceptive care that facilitates access to the full range of contraceptive options is crucial to realizing reproductive autonomy and reproductive justice.

## FUNDING INFORMATION

California Academy of Family Physicians (CAFP) Student Research Grant to RLN to support this project. RLN is supported by National Institute of General Medical Sciences Medical Scientist Training Program (T32GM007618). Research grant to JK from UCSF Family and Community Medicine (FCM).

## CONFLICT OF INTEREST STATEMENT

The authors declare no conflicts of interest.
